# Histamine H4 receptor regulates IL-6 and INF-γ secretion in native monocytes from healthy subjects and patients with allergic rhinitis

**DOI:** 10.1186/s13601-019-0288-1

**Published:** 2019-09-30

**Authors:** Hua Peng, Jian Wang, Xiao Yan Ye, Jie Cheng, Cheng Zhi Huang, Li Yue Li, Tian Ying Li, Chun Wei Li

**Affiliations:** 1 Department of Otorhinolaryngology, Guangzhou General Hospital of Guangzhou Military Command, Guangzhou, China; 2 Department of Otolaryngology, The First Affiliated Hospital, Sun Yat-sen University, No. 58, Zhong Shan 2nd Street, Guangzhou, 510080 China; 3 Guangzhou Key Laboratory of Otorhinolaryngology, The First Affiliated Hospital, Sun Yat-sen University, Guangzhou, China

**Keywords:** Histamine H4 receptor, Histamine H1 receptor, Allergic rhinitis, Native human monocyte, Th1 and Th2 cytokines

## Abstract

Histamine H1 receptor (H1R) and histamine H4 receptor (H4R) are essential in allergic inflammation. The roles of H4R have been characterized in T cell subsets, whereas the functional properties of H4R in monocytes remain unclear. In the current study, the responses of H4R in peripheral monocytes from patients with allergic rhinitis (AR) were investigated. The results confirmed that H4R has the functional effects of mediating cytokine production (i.e., down-regulating IFN-γ and up-regulating IL-6) in cells from a monocyte cell line following challenge with histamine. We demonstrated that when monocytes from AR patients were stimulated with allergen extracts of house dust mite (HDM), IFN-γ secretion was dependent on H4R activity, but IL-6 secretion was based on H1R activity. Furthermore, a combination of H1R and H4R antagonists was more effective at blocking the inflammatory response in monocytes than treatment with either type of antagonist alone.

## To the editor

Allergic rhinitis (AR) has long been considered to be mainly mediated by activation of histamine H1 receptor (H1R) [1], although the use of histamine H1R antagonists to treat this disease has produced unsatisfactory outcomes. Recently, new drugs targeting histamine H4 receptor (H4R) have been applied in the treatment of allergic diseases [2, 3], and a combination of H1R and H4R antagonists is more effective at alleviating allergic symptoms than either type of antagonist alone. The roles of H4R in allergic diseases may contribute to promoting the Th2 immune response in T cell subsets and mast cells [4, 5]. Several reports have suggested the functions of H4R in monocyte-derived macrophages and dendritic cells in atopic dermatitis [6, 7]; in addition, two studies have indicated the functional roles of H4R in native monocytes from healthy subjects by inhibiting CCL2 production or IL-12p70 secretion [8, 9]. However, limited information is available regarding H4R in peripheral monocytes from allergic patients (such as AR). Therefore, the current study sought to investigate the functional responses of H4R to histamine, allergen extracts (*Dermatophagoides pteronyssinus and Dermatophagoides farinae*) of house dust mite (HDM) and histamine agonists/antagonists in monocytes from AR and healthy subjects.

Cells from the U937 human monocyte cell line, primary monocytes from healthy subjects (n = 12) and primary monocytes from AR subjects (n = 12) were used to study the functional properties of H4R in monocytes in response to histamine, a selective H4R agonist (4-MeHA), HDM, an H1R antagonist (triprolidine) and an H4R antagonist (JNJ7777120). Functional responses were evaluated based on secretion of the Th1 cytokine IFN-γ and the Th2 cytokine IL-6 upon treatment with a stimulant or inhibitor. Details regarding the experimental materials and methods are provided in Additional file 1.

**Fig. 1:**
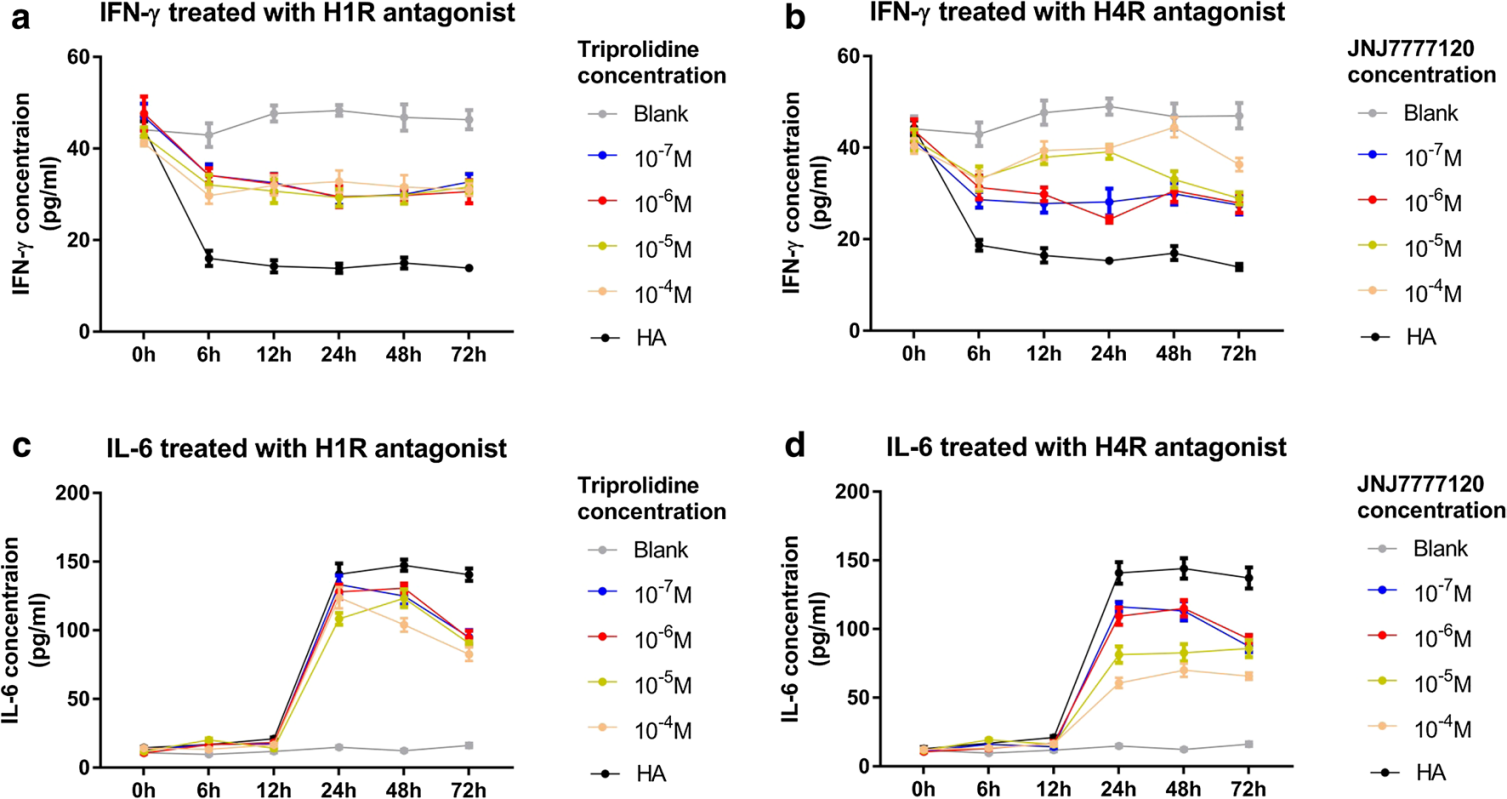
IFN-γ and IL-6 secretion upon treatment with H1R or H4R antagonist. IFN-γ (**a**, **b**) and IL-6 (**c**, **d**) production was observed in U937 cells, which were challenged by histamine first and then treated by H1R (triprolidine) or H4R (JNJ7777120) antagonist under different dose and time points. Data are shown as the mean ± SEM

H4R mRNA expression was evident in U937 monocytes, for which H4R mRNA levels were higher than H1R mRNA levels (Additional file 2: Figure S1). To optimize the stimulation conditions, U937 cells were treated with histamine or 4-MeHA at different concentrations (10^−9^ M to 10^−4^ M) and were observed at multiple time points (0, 6, 12, 24, 48, and 72 h). The results showed that the down-regulation of IFN-γ and the up-regulation of IL-6 were both dose- and time-dependent (Additional file 2: Figure S1). The optimal conditions involved monocyte stimulation with 10^−5^ M of histamine or 4-MeHA for 24 h (Additional file 2: Figure S1). To evaluate the potencies of the H1R antagonist (triprolidine) and the H4R antagonist (JNJ7777120), dose–response experiments (for doses of 10^−9^ M to 10^−4^ M) were performed in histamine-treated cells, and observations were obtained at various time points (0, 6, 12, 24, 48, and 72 h). Dose curves showed that JNJ7777120 could regulate cytokine secretion in a dose-dependent manner, whereas neither IFN-γ nor IL-6 exhibited significantly altered concentrations as triprolidine doses increased (Fig. 1). In addition, the results indicated that for evaluating the effects of H1R and H4R antagonists, the optimal concentration and time point were 10^−5^ M and 24 h, respectively.

**Fig. 2:**
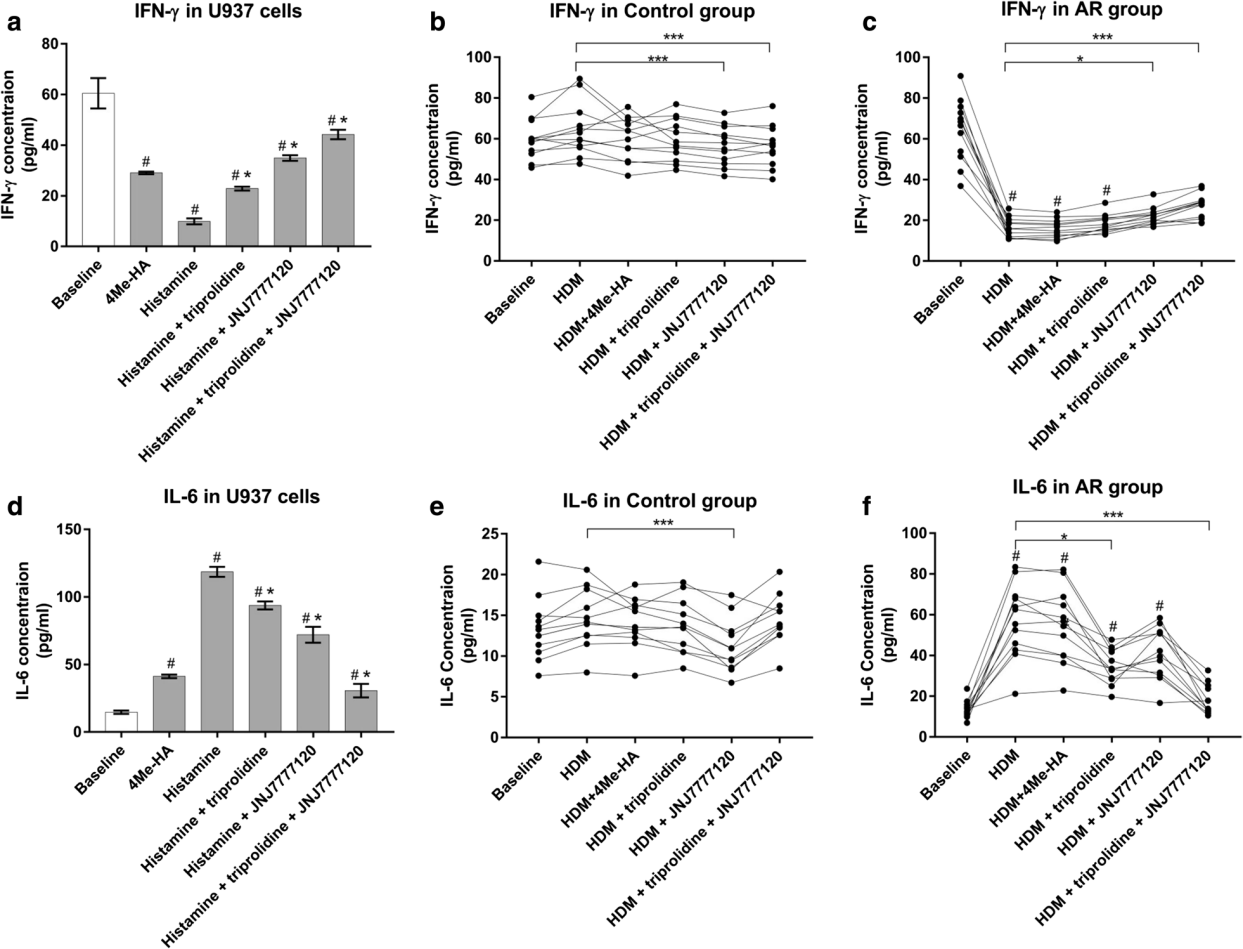
Blockage effects of H1R, H4R antagonists in U937 cells and human native monocytes with regard to IFN-γ and IL-6 secretion. IFN-γ and IL-6 levels were compared in U937 cells treated with H1R or H4R antagonist alone or combined H1R and H4R antagonists after histamine stimulation (**a**, **d**). IFN-γ and IL-6 levels were compared in native monocytes from healthy subjects (**b**, **e**) or allergic rhinitis patients (**c**, **f**) by treating with H1R or H4R antagonist alone or combined H1R and H4R antagonists after house dust mite (HDM) stimulation. Data are shown as the mean ± SD. ^#^*p* < 0.05 versus baseline. *p < 0.05, **p < 0.01, ***p < 0.001 versus histamine-stimulated or HDM-stimulated cells

The cytokine-blocking effects of H1R antagonist, H4R antagonist, and combined H1R and H4R antagonist treatment were further compared under the same optimized conditions (a stimulant concentration of 10^−5^ M and a time point of 24 h). IFN-γ production was suppressed by histamine but gradually enhanced by treatment with triprolidine (2.3-fold), JNJ7777120 (3.5-fold), and the combination of the H1R and H4R antagonists (4.5-fold). IL-6 levels were increased by histamine but progressively down-regulated by treatment with triprolidine (− 1.2-fold), JNJ7777120 (− 1.6-fold), and the combination of the H1R and H4R antagonists (− 3.8-fold) (Fig. 2). Although the two antagonist compounds exhibited synergistic effects when used together, this combination could not completely block the histamine-induced cytokine response (i.e., cytokine levels were not normalized to those of non-stimulated monocytes), indicating that other histamine receptors may remain active in this response.

The H4R-related secretion properties of native monocytes from AR patients and healthy controls in response to HDM, the H4R agonist, the H1R antagonist and the H4R antagonist were investigated. Following HDM stimulation, IFN-γ and IL-6 were significantly decreased and elevated, respectively, in AR patients but not in healthy subjects (Fig. 2); however, the H4R agonist did not enhance the cytokine response in human monocytes challenged with HDM. In monocytes from AR patients, IFN-γ down-regulation was partially reversed by treatment with the H4R antagonist (1.4-fold, p < 0.05) or the combination of the H1R and H4R antagonists (1.7-fold, p < 0.001) but remained unchanged upon treatment with the H1R antagonist alone (Fig. 2). In contrast, in these monocytes, IL-6 up-regulation was partially blocked when the H1R antagonist (− 1.6-fold, p < 0.05) or the combination of the H1R and H4R antagonists (− 3.5-fold, p < 0.001) was administered but remained unchanged upon treatment with the H4R antagonist alone (Fig. 2). These results demonstrated discrepancies in the characteristics of the cytokine-blocking effects produced by the H1R and H4R antagonists in AR patients.

In the present study, the potencies of H4R and H1R antagonists following stimulation by histamine or HDM in cells from a monocyte cell line and native human monocytes from AR and control subjects have been compared for the first time. Histamine or HDM could modulate the secretion of IFN-γ and IL-6 by monocytes, and our results confirmed that cytokine-blocking effects were observed upon treatment with an H4R- or H1R-specific antagonist. These findings indicated the functional properties of H4R and H1R in monocytes with respect to mediating the secretion of Th1 and Th2 cytokines. A previous study reported that peripheral monocytes were important sources of histamine in blood other than basophils, and histamine could be released in monocytes by stimulation with specific antigens [10]. Our study also found that the cytokine secretion in monocytes from AR patients was not fully blocked by treatment with H1R and H4R antagonists. The above evidence could be due to interference by other pleiotropic mediators released by stimulated monocytes, e.g., histamine could be induced by HDM and bind to histamine receptors in an autocrine fashion to modulate cytokine production. Interestingly, the current study suggested different blocking effects of H4R and H1R antagonists in monocytes from AR patients, showing that after HDM stimulation, H4R and H1R antagonists could up-regulate IFN-γ production and attenuate IL-6 levels, respectively. In addition, the results demonstrated that when used together, H4R and H1R antagonists act in synergy to block the allergic reaction in cells from a monocyte cell line and in monocytes from AR patients. Therefore, these results suggest that H4R and H1R may have an intrinsic relationship in the context of mediating histamine- or HDM-induced allergic response. Furthermore, our findings also indicated that therapeutic strategies should target both H4R and H1R, which may yield more beneficial effects for AR-related inflammation compared with strategies that focus on only one type of histamine receptor. In conclusion, we showed that H4R is expressed by monocytes and mediates Th1 and Th2 cytokine secretion in these cells. Therefore, combining H4R and H1R antagonism should produce synergistic benefits in chronic allergic conditions such as AR.

## Supplementary information

**Additional file 1.** Supplemental materials and methods.

**Additional file 2: Figure S1.** Dose and time point experiments for secretion of IFN-γ and IL-6 in U937 cells upon histamine or 4-MeHA stimulation. The relative mRNA expression levels of H1R and H4R in U937 cells were analyzed by quantitative PCR (A). Data shown as mean ± SD. Secretion of IFN-γ and IL-6 was shown following the time series (from 0 h to 72 h) in different concentrations of histamine (B, C) or 4-MeHA (D, E). Data shown as mean ± SEM.

## Data Availability

The datasets used and/or analysed during the current study are available from the corresponding author on reasonable request.

## Abbreviations

H1R: histamine H1 receptor
H4R: histamine H4 receptor
AR: allergic rhinitis
HDM: house dust mite
