# Supplementary material for: Histamine H4 receptor regulates IL-6 and INF-γ secretion in native monocytes from healthy subjects and patients with allergic rhinitis
Source: Clin Transl Allergy. 2019 Sep 30;9:49. doi: 10.1186/s13601-019-0288-1 (PMC6767641; doi:10.1186/s13601-019-0288-1)
Supplement: Supplementary file 1 — Additional file 1. Supplemental materials and methods. [file 13601_2019_288_MOESM1_ESM.docx]

**Additional file 1**

**Histamine H4 receptor regulates IL-6 and INF-γ secretion in native monocytes from healthy subjects and patients with allergic rhinitis**

**Materials and Methods**

**Culture of U937 cells**

Human U937 cell lines were purchased from the American Type Culture Collection (ATCC, USA). Cells were cultured in RPMI 1640 medium (GIBCO, USA) supplemented with 10% foetal bovine serum, streptomycin (100 U/ml) and penicillin (100 U/ml) at 37°C in a humidified atmosphere with 5% CO_2_. The culture medium was changed every 3 days. To assess the regular functions of H4R in monocytes, U937 cells were suspended again in RPMI 1640 medium to adjust cell density to 1×10^5^ cells per ml and were then transferred into 24-well round-bottom plates (500 µl/well).

**Characteristics of AR patients and healthy subjects**

Twelve patients (6 males, 6 females; mean age 28 years) suffering from persistent AR with moderate to severe nasal symptoms (e.g., nasal itching, sneezing, nasal obstruction and rhinorrhoea) were recruited at a clinic in First Affiliated Hospital of Sun Yat-sen University. All patients were diagnosed by an ENT/allergy specialist in accordance with ARIA criteria. The inclusion criteria for the AR patients were (1) an age>18 years; (2) persistent AR symptoms during the prior two years; (3) allergic sensitization to *Dermatophagoides pteronyssinus* and *Dermatophagoides farina*, as confirmed by a serum sIgE test; (4) no prior treatment with allergen-specific immunotherapy; and (5) negative skin prick test results to other common inhalant allergens (e.g., common pollens, cockroaches, fungi, and animal dander). Serum sIgE was assessed using ImmunoCAP tests (Phadia, Uppsala, Sweden), and a value of more than 0.35 kUA/l was regarded as a positive allergic response. In addition, 12 healthy volunteers (7 males, 5 females; mean age 23 years) without any allergy history or nasal symptoms were selected as a control group. The suitability of the control subjects was further verified by obtaining negative SPT results for all common inhalant allergens for these individuals. No recruited subjects (AR patients or controls) suffered from an infection, asthma, an autoimmune disease or an upper airway disease other than AR, such as septal deviation, nasal polyps, or sinusitis, prior to enrolment. This study was approved by the ethics committee of First Affiliated Medical Hospital of Sun Yat-sen University in Guangzhou, China.

**Monocyte enrichment and isolation**

Whole blood (about 8ml) was obtained from the AR patients (n=12) and healthy subjects (n=12) and the buffy coats were collected. Monocytes were isolated from the buffy coats by using a density gradient centrifugation method with OptiPrep™ reagents (Axis-shield, Norway) following the manufacture guideline. The isolated monocytes have been analyzed by flow cytometry with evaluation of CD14+ cells and the purity of the monocytes was more than 90%. Cell viability (≥95%) was confirmed by trypan blue staining under the microscope. Isolated monocytes were cultured in RPMI 1640 medium supplemented with streptomycin (100 U/ml), penicillin (100 U/ml) and 10% FBS.

**Chemicals and stimulants**

Histamine, H1R antagonist (triprolidine), H4R antagonist (JNJ7777120) and H4R agonist (4-MeHA) were purchased from Sigma Aldrich (Darmstadt, Germany). Preparation of the chemicals was re-suspended in phosphate buffer solution (PBS) in 10^-3^ M concentration; and then they were serially diluted into 10^-4^ , 10^-5^, 10^-6^, 10^-7^, 10^-8^, 10^-9^ M for experimental use. Allergen mixture is purified natural allergens (50% *Dermatophagoides pteronyssinus* and 50% *Dermatophagoides farinae*) (INDOOR biotechnologies, Charlottesville, VA) and the concentration used in the current study was 50 μg/ml.

**Quantitative real-time PCR (qRT-PCR)**

Total RNA was extracted from U937 cells by using Trizol reagent according to the manufacturer’s guidelines (Invitrogen, USA). RNA was reversely transcribed and real-time RT PCR experiment was performed by using ABI 7300 PCR system (Applied Biosystems, Foster City, CA). Real-time PCR was performed with Quantitect Primer Assays (Qiagen, Germany) for H1R (NM_000861), H4R (NM_021624) and glyceraldehyde 3-phosphate dehydrogenase (GAPDH) (NM_002046.3) using SYBR Green detection method. The assay information was as follows: Hs_HRH4_1_SG, QuantiTect Primer Assay (#QT00032326); Hs_HRH1_1_SG, QuantiTect Primer Assay (#QT00199857); Hs_GAPDH_1_SG, QuantiTect Primer Assay (#QT00079247). The relative gene expression of H4R and H1R was calculated by using the comparative C_t_ (ΔC_t_) method, with GAPDH as a reference.

**Measurement of IFN-γ and IL-6 secretion in U937 cells and the primary monocytes treated with different stimulants**

U937 cells were treated with gradient concentrations (10^-9^M to 10^-4^M) of histamine, H1R antagonist (triprolidine), H4R antagonist (JNJ7777120), and histamine agonist (4-MeHA). Supernatants were harvested before stimulation and 6h, 12h, 24h, 48h, 72h after stimulation. Human monocytes from AR patients and control subjects were treated with histamine, H1R antagonist (triprolidine), H4R antagonist (JNJ7777120), histamine agonist (4-MeHA), and HDM at optimized concentration. Supernatants were harvested before stimulation and 6h, 12h, 24h, 48h, 72h after stimulation. The cytokine production levels of IFN-γ and IL-6 in supernatants were analyzed by enzyme-linked immunosorbent assay (ELISA) kits (R&D, Minneapolis, MN). The lower detection limit of IFN-γ and IL-6 assays was 8 pg/mL & 0.7 pg/mL, respectively.

**Statistical analysis**

All data were analyzed using the SPSS statistical software V18.0 (SPSS Inc., Chicago, IL). The t-test was used to compare the mRNA of U937 cell difference between H4R and H1R. Repeated-measures Anova was used to determine the statistical significance of the cytokines in supernatant within groups (different time or dose). Paired t-test, Two-factor Anova, One-way Anova, and Factor Analysis were used to assess the data in human monocyte. Significance was accepted when p-value < 0.05.
