# Supplementary material for: Histamine H4 receptor regulates IL-6 and INF-γ secretion in native monocytes from healthy subjects and patients with allergic rhinitis
Source: Clin Transl Allergy. 2019 Sep 30;9:49. doi: 10.1186/s13601-019-0288-1 (PMC6767641; doi:10.1186/s13601-019-0288-1)
Supplement: Supplementary file 2 — Additional file 2: Figure S1. Dose and time point experiments for secretion of IFN-γ and IL-6 in U937 cells upon histamine or 4-MeHA stimulation. The relative mRNA expression levels of H1R and H4R in U937 cells were analyzed by quantitative PCR (A). Data shown as mean ± SD. Secretion of IFN-γ and IL-6 was shown following the time series (from 0 h to 72 h) in different concentrations of histamine (B, C) or 4-MeHA (D, E). Data shown as mean ± SEM. [file 13601_2019_288_MOESM2_ESM.docx]

**Additional file 2**

**
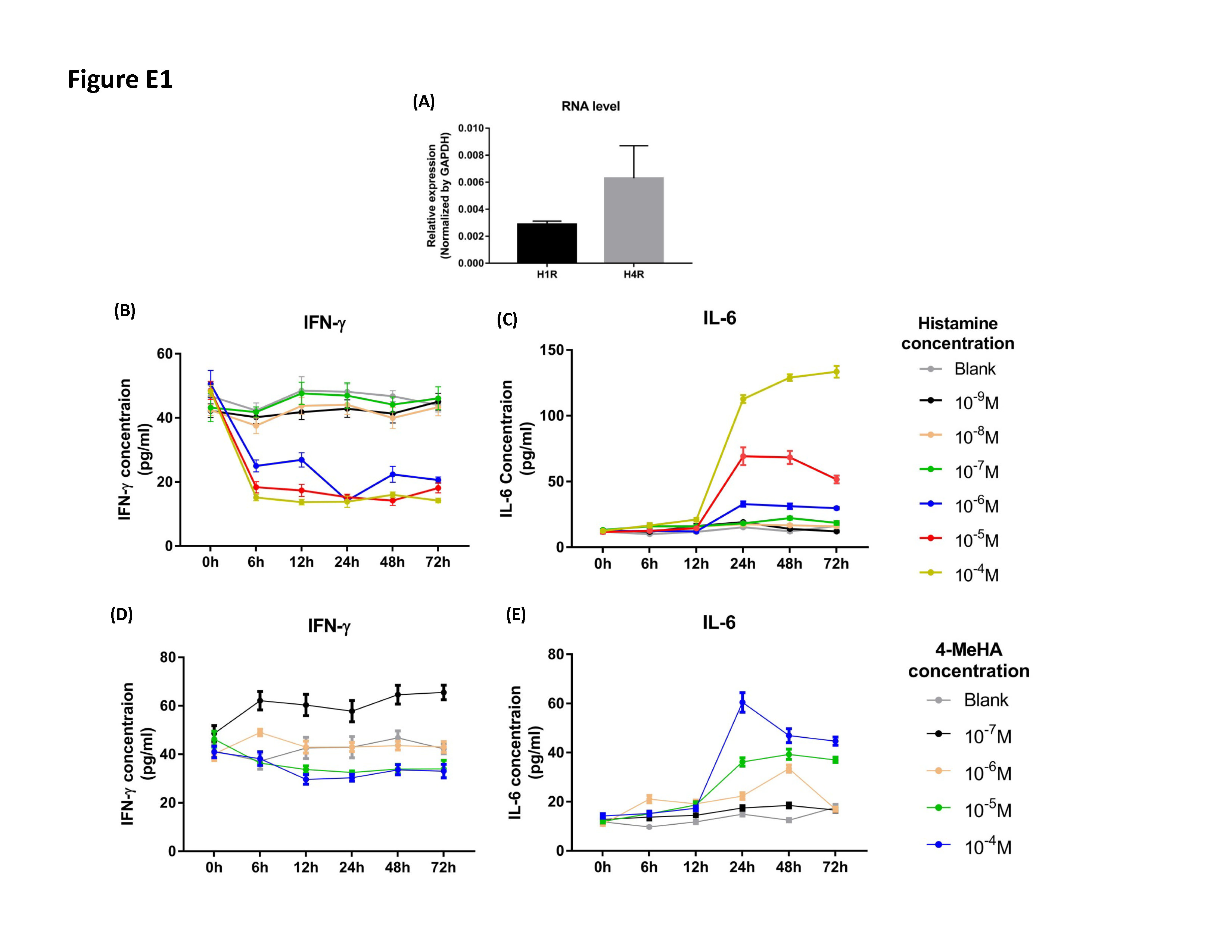
**

**Figure S1.** Dose and time point experiments for secretion of IFN-γ and IL-6 in U937 cells upon histamine or 4-MeHA stimulation. The relative mRNA expression levels of H1R and H4R in U937 cells were analyzed by quantitative PCR (A). Data shown as mean ± SD. Secretion of IFN-γ and IL-6 was shown following the time series (from 0h to 72h) in different concentrations of histamine (B, C) or 4-MeHA (D, E). Data shown as mean ± SEM.
